# Potential Therapeutic Implication of Herbal Medicine in Mitochondria-Mediated Oxidative Stress-Related Liver Diseases

**DOI:** 10.3390/antiox11102041

**Published:** 2022-10-17

**Authors:** Moon Nyeo Park, Md. Ataur Rahman, Md. Hasanur Rahman, Jong Woo Kim, Min Choi, Jeong Woo Kim, Jinwon Choi, Myunghan Moon, Kazi Rejvee Ahmed, Bonglee Kim

**Affiliations:** 1 Department of Pathology, College of Korean Medicine, Kyung Hee University, Hoegi-dong, Dongdaemun-gu, Seoul 02447, Korea; 2 Korean Medicine-Based Drug Repositioning Cancer Research Center, College of Korean Medicine, Kyung Hee University, Seoul 02447, Korea; 3 Global Biotechnology and Biomedical Research Network (GBBRN), Department of Biotechnology and Genetic Engineering, Faculty of Biological Sciences, Islamic University, Kushtia 7003, Bangladesh

**Keywords:** nonalcoholic fatty liver disease (NAFLD), herbal medicine, catechol-containing antioxidants, mitochondria, oxidative stress, liver diseases

## Abstract

Mitochondria are double-membrane organelles that play a role in ATP synthesis, calcium homeostasis, oxidation-reduction status, apoptosis, and inflammation. Several human disorders have been linked to mitochondrial dysfunction. It has been found that traditional therapeutic herbs are effective on alcoholic liver disease (ALD) and nonalcoholic fatty liver disease (NAFLD) which are leading causes of liver cirrhosis and hepatocellular carcinoma. The generation of reactive oxygen species (ROS) in response to oxidative stress is caused by mitochondrial dysfunction and is considered critical for treatment. The role of oxidative stress, lipid toxicity, and inflammation in NAFLD are well known. NAFLD is a chronic liver disease that commonly progresses to cirrhosis and chronic liver disease, and people with obesity, insulin resistance, diabetes, hyperlipidemia, and hypertension are at a higher risk of developing NAFLD. NAFLD is associated with a number of pathological factors, including insulin resistance, lipid metabolic dysfunction, oxidative stress, inflammation, apoptosis, and fibrosis. As a result, the improvement in steatosis and inflammation is enough to entice researchers to look into liver disease treatment. However, antioxidant treatment has not been very effective for liver disease. Additionally, it has been suggested that the beneficial effects of herbal medicines on immunity and inflammation are governed by various mechanisms for lipid metabolism and inflammation control. This review provided a summary of research on herbal medicines for the therapeutic implementation of mitochondria-mediated ROS production in liver disease as well as clinical applications through herbal medicine. In addition, the pathophysiology of common liver disorders such as ALD and NAFLD would be investigated in the role that mitochondria play in the process to open new therapeutic avenues in the management of patients with liver disease.

## 1. Introduction

Mitochondria are double-membrane organelles that participate in a wide range of physiological functions within cells. These functions include cell survival, proliferation, and migration. Mitochondria are essential organelles for the survival of eukaryotes because they contribute to the respiratory adenosine triphosphate process (ATP) [1], due to mitochondrial protein translation and various cellular processes such as free radical generation, calcium homeostasis, cell viability, and apoptosis [2]. Biogenesis of mitochondrial mass is critical in maintaining energy homeostasis during energy deprivation and mitochondrial insults [3,4]. During the oxidative phosphorylation process that provides electron lead to ATP syntheses, the mitochondrial respiratory process subsequently generates radicals and other reactive oxygen species known as ROS [5]. Although mitochondrial ROS are important, they are not the only source of ROS. In this review, we focused on determining and understanding the stress of mitochondrial oxidation caused by an imbalance between oxidants and antioxidants that could serve as a framework for the therapeutic benefits of clinical trials for disease treatment [6].

In recent years, scientists have paid more attention to herbal medicines, which include plants, herbal complexes, and biological ingredients [7]. This is because herbal drugs have a lot of potential to treat diseases, including cancer and oxidative stress [8]. In the past few decades, medicinal herbs and their bioactive parts have been used successfully to treat different types of cancer as a supplement to standard treatments such as chemotherapy, radiation therapy, targeted therapy, or immunotherapy [9]. Numerous herbal products made from these herbs have been shown to stop the growth of cancer cells and mitochondria stress with fewer side effects than traditional cancer treatments [10,11]. In this study, we focus on the use of herbal bioactive elements as an adjuvant therapy against a variety of mitochondria-mediated oxidative stress-related liver diseases. The regulation of mitochondrial function by these substances has been a primary focus of our research since it has the potential to contribute to a deeper and more comprehensive understanding of novel approaches to liver disease via mitochondria-mediated oxidative stress. As a means of doing this, we carried out a literature analysis using molecular pharmacology with the intention of deciphering herbal medicine with therapeutic targets for mitochondria-related oxidative stress in hepatotoxicity to control liver disease.

## 2. Production of Reactive Oxygen Species (ROS) Due to Oxidative Stress

**Figure 1:**
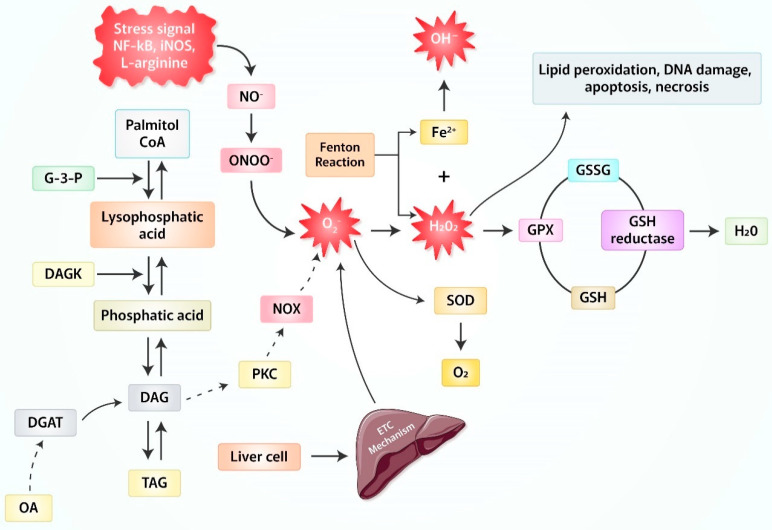
ROS production from oxidative stress. Locations at which ROS are produced. There are several distinct locations within a cell that are capable of producing ROS. The vast majority of them may be found in the mitochondrial surroundings. Glyceraldehyde-3-phosphate (GAPDH), diacylglycerol:acyltransferase (DGAT), diacylglycerol (DAG), triacylglycerol (TAG), peroxynitrite (ONOO^−^), nitric oxide (NO), protein kinase C (PKC), glutathione peroxidase (GPx), superoxide dismutase (SOD), glutathione (GSH), GSH/oxidized glutathione (GSSH), electron transport chain (ETC), inducible nitric oxide synthase (iNOS).

The term Reactive Oxygen Species (ROS) refers to a variety of reactive molecules and free radicals formed from molecular oxygen. Recent research has demonstrated that ROS play an important part as a messenger in the regular process of cell cycling and signal transduction inside cells. It is generated by the univalent reduction of molecular oxygen. This reaction is caused by a catalyst from an enzyme called nicotinamide adenine dinucleotide phosphate dehydrogenase (NADPH) and xanthine oxidase (XOD). ROS are involved in many biological functions (Figure 1). High amounts of ROS can cause cellular damage, oxidative stress, and DNA damage, depending on severity and length of exposure. Nitric oxide anion (NO•) acts as a cell-to-cell messenger, lowering blood pressure. ROS species and antioxidant enzymes may switch enzymes on and off intracellularly through redox signaling, similar to the cAMP second messenger pathway. Superoxide anion and hydroperoxide are examples. O•^2−^ has a low steady-state level, limiting its spatial activity. Hydrogen peroxide (H_2_O_2_) is unreactive with thiols in the absence of catalytic agents (e.g., enzymes, multivalent metals, etc.). However, it reacts with thiolate anion (S^−^) to generate sulfenic acid, which ionizes to form sulfenate (SO^−^). Glutathione reverses this intermediary.

ROS secreted from mitochondria is removed by cell antioxidant systems, and various cell components are oxidatively damaged due to hydroxyl radical (•OH) formation [12,13]. Low and moderate level of ROS is a critical mediator of metabolism and inflammation, but an excessive level of ROS contributes to apoptosis or autophagy containing H_2_O_2_^−^ sensitive pathways, respectively [14,15,16,17]. Notably, excessive amounts of ROS are highly toxic to cells. Oxidative stress causes pathogenesis of various degenerative diseases, such as diabetes, cancer, cardiovascular disorders or neurodegenerative diseases due to their effects on lipid, proteins and DNA [18]. This high mutation rate is due to the presence of mitochondrial genomes close to the production site of free radicals without including intone or histone, which prefer a higher amount of deoxyguanosine triphosphate (dGTP) than other deoxynucleoside triphosphates (dNTPs), and replication after asymmetric division [19]. As referred to above, oxidative stress due to mitochondrial dysfunction is an important factor in non-alcoholic steatohepatitis (NASH) and alcoholic steatohepatitis (ASH), known as the origin of steatohepatitis (SH), and contributes to other disease-related mechanisms (e.g., vesicle endoplasmic reticulum (ER) stress, and autophagy disorder). However, NASH is primarily recognized as mitochondrial disease [20,21,22].

## 3. Oxidative Stress-Related Mitochondrial Reactive Oxygen Species (ROS)/Signaling in Liver

Liver is a major mediator such as metabolism, synthesis, carbohydrates, vitamins, and lipids, and is a place for high metabolic activity related to free oxygen production [23]. Diamine oxidase, aldehyde dehydrogenase, tryptophan double oxidase, liver dehydrogenase, and cytochrome P450 enzyme system are enzymes that induce active oxygen in the liver [23,24].

Additionally, mitochondria and ER can generate ROS in the liver through the cytochrome P450 enzyme, which is formed by macrophages and neutrophils [25,26]. Mitochondria are the main place of oxygen consumption, and the generation of ROS is caused by oxygen consumption in the mitochondrial respiratory chain (MRC) [27]. 

**Figure 2:**
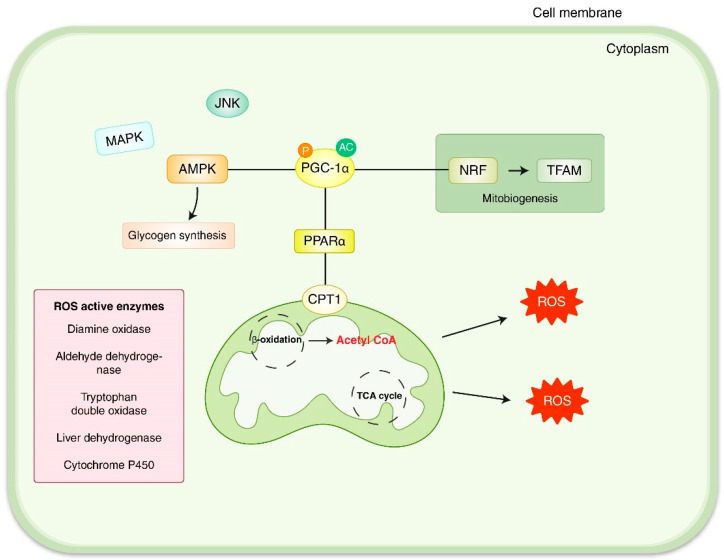
Role of mitochondrial dysfunction in ROS production. Mitogen-mediated protein kinases (MAPKs), adenosine monophosphate-mediated protein kinase (AMPK), Peroxisome proliferator-activated receptor gamma coactivator 1-alpha (PGC-1α), Peroxisome proliferator-activated receptor gamma (PPARγ), carnitine palmitoyltransferase-1 (CPT-1), c-Jun N-terminal kinase (JNK), nuclear factor erythroid 2-related factor 2 (Nrf2), mitochondrial transcription factor A (TFAM).

The dual role of ROS/oxidative stress in signaling pathways can determine the final role of mitochondrial dysfunction as a cause or consequence of disease progression. It was suggested that restrictions or impairments to the action of antioxidants could lead to an accumulation of ROS that could have harmful effects in cell functions including aging or liver disease [6]. ROS originated from mitochondria, which activate adenosine monophosphate-mediated protein kinase (AMPK) [28,29] and mitogen-mediated protein kinases (MAPKs), such as c-Jun N-terminal kinase (JNK) [16]. AMPK facilitate glucose and fatty acid β-oxidation and consecutively stimulate peroxisome proliferator-activated receptor gamma coactivator 1-alpha (PGC-1α). Peroxisome proliferator-activated receptor gamma (PPARγ) is activated by PGC-1α, which induces fatty acid-metabolizing enzymes including carnitine palmitoyltransferase-1(CPT-1) and acyl Co-A dehydrogenase (ACADs), lead to β-oxidation of fatty acid in mitochondria (Figure 2) [30,31]. PGC-1α also plays a pivotal role in the increase in mitochondrial mass and mitochondrial respiratory capacities through the regulation of nuclear factor erythroid 2-related factor 2 (Nrf2) and AMPK [32,33,34]. Carbohydrates’ catabolism produces a high level of glucose and insulin and is associated with hepatic-free fatty acid (FFA) synthesis [21,35,36]. Hepatic β-oxidation causes FFA, also known as non-esterified fatty acids (NEFA) to be acetyl-CoA, which is a process of generating energy in a healthy state in which it is completely CO_2_ by the Krebs circuit [33]. 

## 4. Liver Impairment Mediated by Mitochondrial Reactive Oxygen Species (ROS) Generation 

Liver is an important organ that requires high energy for the secretion of polysynthesis and endogenous compounds, and liver disease is closely related to mitochondrial dysfunction [37].

As mentioned earlier, mtDNA encodes 13 respiratory chain subunits such as complexes I, III, IV, and V which lead to production of ATP and ROS [38]. Once ROS are stimulated, the mitochondrial DNA (mtDNA) is damaged, which can increase ROS, and can amplify oxidative stress by encoding insufficient subunits of the respiratory system, leading to cell death [39]. Chronic liver diseases are regarded as a liver disorder regardless of the cause of the liver disorder due to increased oxidative stress. There are redox-sensitive transcription factors such as early growth response protein 1 (Egr-1), Nuclear factor kappa-light-chain-enhancer of activated B cells (NF-kappaB) and activator protein 1 (AP-1) and G protein-coupled receptor (GPCR), as were essentially involved in mitogen-activated protein kinase events [40]. As mentioned above, it has been reported that hepatocytes lead to apoptosis by oxidative-dependent chain reaction in the liver. However, the oxidative-dependent cellular process has not been fully elucidated so far. This redox reaction increased rapidly in redox state and previous reports confirmed that there was a relation between oxidants and expression of apurinic/apyrimidinic endonuclease (APE)/redox factor (Ref)-1 [41,42]. Once exposed to oxidative stress, antioxidant-related genes are activated through a protection mechanism in the reactive antioxidant response element (ARE) [43]. Stress-activated transcription factor Nrf2 induces a defensive mechanism against oxidative stress damage, and emerging evidence deems this signaling pathway to be a key pharmaceutical target for the treatment of liver disorders [44]. It was reported that orientin had a role in the amelioration of liver damage by lowering oxidative stress. This suppression of oxidative stress may be closely connected to the activation of Nrf2/ARE, which occurs through the phosphatidylinositol 3-kinase/protein kinase B (PI3K/Akt) and P38/MAPK signal pathways [45]. Additionally, using the Nrf2 and NF-κB signaling pathways, a polysaccharide called PFP-1 from the *Pleurotus geesteranus* fungus can reduce the severity of alcoholic liver disorders [46]. However, ARE-containing gene is extensively controlled by Nrf2 in association with glutathione (GSH) homeostasis, NAD(P)H quinone oxidoreductase 1 (NQO1), and UDP-glucosyltransferase (UDP) [47,48]. Multiple causes of chronic liver disease result in inflammatory responses and necrosis, which destroy liver tissue and lead ultimately to liver cirrhosis [49,50]. Liver fibrosis is a clinical stage of nearly all chronic liver illnesses preceding cirrhosis, and its histology is characterized by excessive accumulation of extracellular matrix and inflammatory responses that interfere with the normal liver function [51]. 

## 5. Source and Defense System for Mitochondria-Mediated Oxidative Stress and Reactive Oxygen Species (ROS) Production in Liver Disease

**Figure 3:**
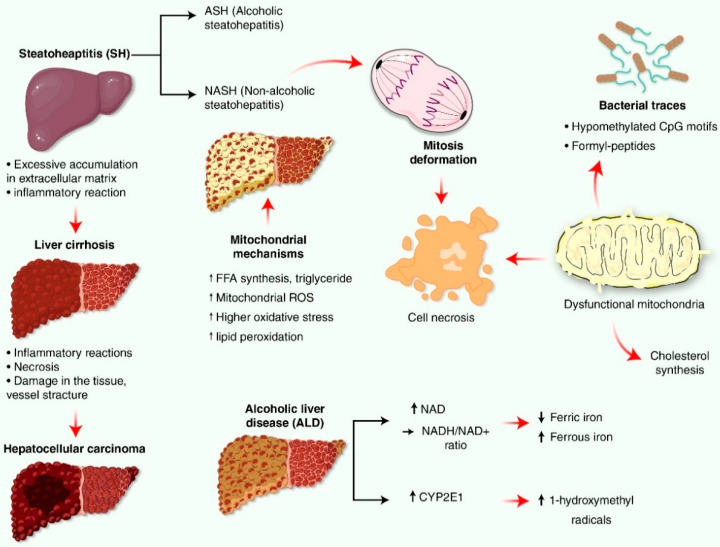
The pathogenesis of NAFLD-related hepatocellular carcinoma is depicted here in the form of a diagram. Steatohepatitis (SH), alcoholic steatohepatitis (ASH), non alcoholic steatohepatitis (NASH), alcoholic liver disease (ALD), free fatty acid (FFA), cytochrome P-450 2E1 (CYP2E1), nicotineamide adenine dinucleotide. NASH disrupt the mitochondrial pathway of the liver, in an increase in FFA flow to the liver, mitochondrial ROS, oxidative stress and lipid peroxidation. Due to the accumulation of severely damaged and dysfunctional mitochondria, deformed mitosis leads to cell death. This is caused by the release of bacterial traces (hypomethylated CpG motifs and formyl-peptides) stored in mitochondria, which can speed up the progression of hepatitis and NASH. The dinucleotide NAD+ raises the ratio of NADH to NAD+, resulting in steatosis. Increased CYP2E1 activities result in increased hydroxyl radicals, which is linked to the development of ALD. ↑, up-regulation; ↓, down-regulation.

The free group and the active oxygen may be produced by various enzymes in the cytoplasm, such as amino acid oxidase, cyclooxygenase, lipoxygenase, nitric oxide (NO) generating enzyme, and xanthine oxidase anion [52]. These enzymes are linked to the production process of ROS involved in pathogenesis, and cyclooxygenase and lipoxygenase are associated with arachidonic acid metabolism and inflammation along cancer [53,54], which have a significant effect on the production of peroxide anions, whereas xanthin reperfusion associated with peroxide anions. Oxidants are produced not only by protein secretion, but also by sulfur hydryl oxidase in ER during protein folding and disulfide bond formation in peroxide by peroxide oxidase [55,56,57]. Hepathology is known to be due to peroxide anions caused by nicotine amide adenine dinucleotide phosphate as electrons are transferred to molecular oxygen in NADPH [58]. The mitochondrial hydrogen peroxide is decomposed by antioxidant enzymes such as peroxiredoxins (Prx) and reduced GSH peroxidases (GPx), including Gpx1 and Gpx4. In addition, Grx2 in the mitochondrial matrix catalyzes protein thiol, oxidized GSH, glutathionylated protein, and thiol-sulfide between oxidized GSH (GSSG). Hence, Grx was considered to play an important role in optimal protein activity in mitochondria [59,60]. Most mitochondria are deficient in catalase, so mitochondrial GSH (mGSH) pools play an important role in removing hydrogen peroxide. mGSH also plays a key role in detoxifying hydroperoxides present in phospholipid sources [59,60]. The imbalance in free ROS and electron-beneficial antioxidant defenses has been the basis for the use of antioxidants as potential treatments for the treatment of human fatty liver disease, and as a result, there have been many experiments testing the role of antioxidant therapy in NAFLD and ALD [6] (Figure 3). Oxidative stress, inflammation, fibrosis, and liver cancer are associated. The role of free radicals on inflammation, fibrosis, and liver cancer of plant-derived antioxidants on proinflammatory signaling pathways, such as NF-kappaB/NLRP3 inflammasome, are important in liver disease. Chronic oxidative stress and inflammation cause liver cirrhosis. NOX4 and NLRP3 are emerging as liver fibrosis therapy targets. Baicalin (BA), a natural flavone, reduced hepatic NLRP3 inflammasome components, NLRP3 and caspase-1, which activate interleukins (IL), measured as IL-1. BA reduced NF-B-driven hepatic inflammation via IL-6 [61]. 4-Acetylantroquinonol B (4-AAQB) improved ALT, AST, and NAFLD activity score (NAS) in MCD-fed mice. 4-AAQB decreased inflammatory responses, ER stress, and NLRP3 inflammasome activation, but elevated Nrf2 and SIRT1 signaling pathways in vitro and in vivo [62]. By inhibiting the NF-B/NLRP3 signaling pathway, kinsenoside is able to reduce fibrosis and inflammation in experimental NASH mice [63]. Melatonin is able to alleviate liver fibrosis caused by Txnrd3 knockdown and nickel exposure through the activation of the IRE1/NF-κB/NLRP3 and PERK/TGF-1 axis [64]. Apigenin has been shown to alleviate the symptoms of non-alcoholic fatty liver disease in mice by downregulating the NLRP3/NF-B signaling pathway [65]. In carbon tetrachloride-induced liver fibrosis, alpinitin activates the Nrf2 pathway while suppressing the NLRP3 pathway, which results in alpinitin’s ability to exhibit anti-inflammatory, anti-oxidative, and anti-angiogenic actions [66]. Paeoniflorin protects db/db mice from developing diabetic liver damage by inhibiting TXNIP-mediated activation of the NLRP3 inflammasome [67].

## 6. Oxidative Stress and Reactive Oxygen Species (ROS) in Nonalcoholic Fatty Liver Disease (NAFLD)

NAFLD has emerged as the chronic liver condition with the greatest rate of growth, becoming a major global health issue. From basic steatosis to NASH, NAFLD encompasses a broad spectrum of histological abnormalities in the liver [68,69,70]. It is also important to note that NASH is closely linked to metabolic syndrome, dyslipidemia, type 2 diabetes, and obesity [71].

The development of hepatic fibrosis, which can lead to cirrhosis, end-stage liver disease, and finally hepatocellular cancer (HCC), is now the most significant clinical problem in NASH [68,70]. Despite the high prevalence and clinical importance of NASH, however, there are nowadays no approved therapeutic agents to arrest or reverse the progression of this disease [69,70]. So far, neither the Food and Drug Administration (FDA) nor the European Medicines Agency (EMA) have approved an ultimate treatment for NAFLD/NASH. This restriction is due to the complexity of the pathogenic pathways implicated, the short duration of existing trials, and the possible synergistic (but as-yet unexplored) effects of combination therapy [72]. On the other hand, early detection and tailored therapy of NASH may reduce the numerous repercussions of increasing liver disease (e.g., the economic burden of end-stage liver disease treatment, the necessity for liver transplantation, and the care of patients with HCC). NAFLD increases the risk of extrahepatic consequences, such as cardiovascular disease and cancer, due to the related metabolic connections [73,74]. 

As a main cause, the definition of NAFLD excludes strong alcohol intake, B and C viruses, many medications, Wilson’s disease, and malnutrition. In this context, NAFLD refers to a metabolic dysfunction-associated fatty liver disease (MAFLD) in which hepatic steatosis is linked with at least one of the following three conditions: obesity, diabetes, or insulin resistance.

Comorbidities: overweight/obesity (particularly visceral fat growth), presence of type 2 diabetes mellitus, and indications of metabolic dysregulation [75]. Events that affect the above-mentioned FFA homeostasis pathways in the hepatocytes might lead to the development of NAFLD. Insulin resistance, visceral fat enlargement, sedentary behavior, and a high-calorie diet are all examples of metabolic disorders that might disrupt the FFA pathway. Metabolic stress is linked to persistent inflammation, significant changes in hepatic lipidology, and the buildup of various lipotoxic substances [74,76]. NAFLD is influenced by the environment, the intestinal microbiota, and an abnormal glucose-lipid ratio, metabolic pathways, metabolic inflammation predominantly driven by innate immunological signaling, adipocytokine dysfunction (e.g., tumor necrosis factor (TNF)-α, adiponectin, resistin, and adiponectin) are all associated with metabolic syndrome, leptin, angiotensin II, and coexisting [77,78,79]. Although the eventual use of genetic results in clinical medicine requires more evidence, only a few genetic variations have been studied thus far. PNPLA3 is expressed on the surface of intrahepatocyte lipid droplets and comprises either lipase or lysophosphatidic acyltransferase activity. Carriers of the variation p.I148M are predisposed to NAFLD, liver fibrosis and cirrhosis, and HCC [79,80,81,82]. Recently, researchers discovered that the rs641738 membrane-bound O-acyltransferase domain-containing 7 (MBOAT7) polymorphism influences histological liver damage in alcoholic liver disease, nonalcoholic fatty liver disease, and chronic hepatitis B (CHB) [83]. The most severe NAFLD modifiers are transmembrane 6 superfamily member 2 (TM6SF2) p.E167K and the rs641738 membrane bound-o-acyltransferase domain-containing 7 (MBOAT7) polymorphisms [84]. A glucokinase regulatory protein (GCKR) variant linked to lipid and glucose characteristics may influence fatty liver infiltration. GCKR rs780094 is related to the severity of liver fibrosis and increased blood lipid levels in NAFLD patients [85]. The rs72613567:TA hydroxysteroid 17-β dehydrogenase 13 (HSD17B13) gene variation enhances phospholipids and protects against fibrosis in nonalcoholic fatty liver disease [86]. Few treatment methods in NAFLD target distinct pathways and may be effective on malfunctioning mitochondria. Antioxidants that target mitochondrial O_2_(−)/H_2_O_2_, for example, are one promising strategy for combating NAFLD-related liver inflammation [87,88].

Damaged mitochondria in liver tissue in obese and nonalcoholic fatty liver disease (NAFLD) patients were identified, outer mitochondrial membrane (OMN) was uncoupled, decreased activity, reduced ATP, and high level of ROS and ROS-mediated mtDNA damage [32,89,90,91]. 

These mitochondrial mechanisms include changes in mitochondrial ROS formation and signaling pathway, changes in mitochondrial biosynthesis and mitochondrial levels of GSH, FFA, lipid peroxide products, and changes in TNF [36]. Compared to the early stage of insulin resistance (IR), NASH patients were found to have decreased antioxidant defense capabilities and increased inflammatory activity due to increased oxidative stress, increased lipid peroxidation, and oxidative DNA damage [32], implying that liver and mitochondria are lost in NASH patients when flexibility is acquired in the initial stage of insulin resistance (IR) [92]. Patients with NASH, including obesity and hyperglycemia, and animal models of ASH, changed mitophagy, which was found to be associated with loss of expression of genes regulating autophagy as well as IR and hyperglycemia [92,93,94]. Moreover, the deformation of mitosis results in cell necrosis due to the accumulation of severe damage and dysfunctional mitochondria, which releases bacterial traces (hypomethylated CpG motifs and formyl-peptides) preserved in mitochondria, and can stimulate hepatitis and NASH progression [36]. Increased cholesterol synthesis within mitochondria in the liver of NASH patients, mitochondrial GSH (mGSH) dissipation was found with steatosis [60,95,96], and culturing liver cells and free cholesterol cause apoptosis and necrosis [60]. Its effects lead to the opening of mitochondrial permeability pores, the release of cytochrome c, liver oxidation stress, and ATP dissipation. Other studies have confirmed that free cholesterol is sensitive to TNF and Fas-induced steatohepatitis, and that it is accompanied by cholesterol-mediated mGSH depletion by a lipopolysaccharide (LPS)-induced liver injury [60]. ROS derived from mitochondria oxidizes unsaturated lipids to lipid peroxidation, which alters mitochondrial proteins including mtDNA and MRC complexes, and this effect partially blocks the transfer of electrons in the MRC, resulting in increased formation of O_2_^–^ and ROS adaptive changes [32,97,98,99,100]. In the liver, ROS-mediated release of TNF damages MRC, induces opening of mitochondrial permeability transition pores, thereby separating oxidative phosphorylation, and increasing mitochondrial ROS formation and lipid peroxidation [97]. Consequently, excessive lipid flow toward hepatocytes can disrupt the mitochondrial voltage-dependent anion channel’s dephosphorylation capacity, inner membrane permeabilization, leading to mitochondrial depolarization, decreased ATP synthesis, and loss of antioxidant capacity [101,102]. 

**Figure 4:**
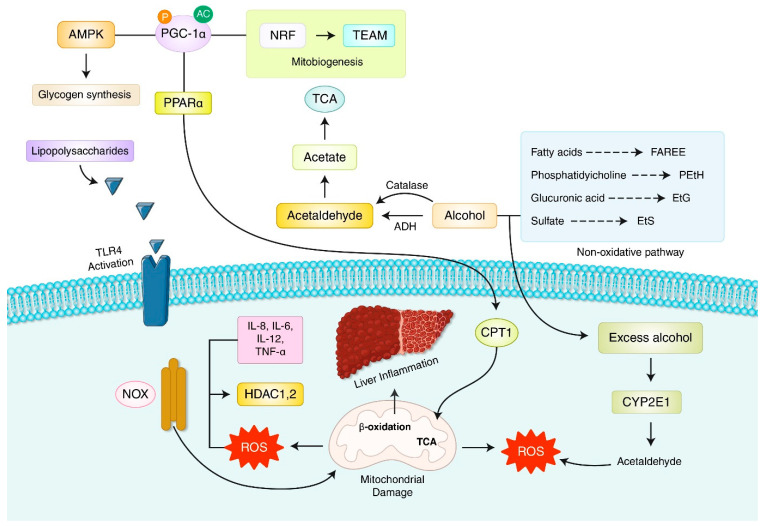
Alcoholic liver disease (ALD) and ROS signaling in liver disease.

Mitochondrial dysfunction mechanisms in progress of steatohepatitis include alcohol abuse, Wilson’s disease, specific drugs, hepatitis B virus (HBV) and hepatitis C virus (HCV) [4,30,31,89,103,104,105,106,107]. The detail mechanism of ALD is explained in Figure 4.

Nuclear factor erythroid 2-related factor 2 (Nrf2), adenosine monophosphate-mediated protein kinase (AMPK), peroxisome proliferator-activated receptor gamma coactivator 1-alpha (PGC-1α), carnitine palmitoyltransferase-1(CPT-1), histone deacetylase (HDAC), NADPH oxidases (NOX), alcohol dehydrogenase (ADH), trichloroacetic acid (TCA), cytochrome P450 2E1 (CYP2E1), peroxisome proliferator-activated receptor gamma (PPARγ), tumor necrosis factor alpha (TNF-α), interleukin-6 (IL-6), interleukin-8 (IL-8), interleukin-12 (IL-12), toll-like receptor 4 (TLR4). 

## 7. Oxidative Stress and Reactive Oxygen Species (ROS) in Alcoholic Liver Disease (ALD)

Alcoholic liver disease (ALD) is a complicated condition globally. Hepatic steatosis, fibrosis, hepatitis, and cirrhosis are all part of the illness spectrum and can all result in the development of hepatocellular carcinoma (HCC). The liver is damaged by excessive alcohol exposure due to two fundamental interconnected processes, oxidative stress and inflammation. An important step in the pathophysiology of ALD is the induction of these two components. There is little doubt that an excessive generation of ROS and the presence of oxidative stress inside hepatocytes contribute to alcohol-induced liver damage. The process of alcohol metabolism in the liver, which starts with alcohol dehydrogenase (ADH), which produces acetaldehyde, may provide an explanation for this mechanism. Acetaldehyde dehydrogenase then converts acetaldehyde to acetate by ALDH. This substance is unstable and quickly decomposes into carbon dioxide and water. Acetaldehyde is a reactive chemical that may react with DNA and build adducts that cause tissue damage, but the creation of acetaldehyde is damaging to liver cells. As they attach to proteins, acetaldehyde and the byproduct malondialdehyde (MDA) create hybrid malondialdehyde-acetaldehyde (MAA) adducts. These substances are identified by scavenger receptors in liver cells, such as Kupffer cells, endothelial cells, and stellate cells, which cause an inflammatory response and the upregulation of cytokines during ALD.

Ethanol metabolism use nicotineamide adenine dinucleotide NAD^+^ to increase the ratio of NADH/NAD^+^ to inhibit mitochondrial β-oxidation, causing steatosis, and inhibiting sirtuin deacetylation and histone deacetylation to damage the epigenetic mechanism of regulating fatty glucose metabolism [97,103,108,109,110]. An increasing ratio of NADH/NAD^+^ reduces ferric iron to ferrous iron, a powerful producer of hydroxy radicals. In addition, the level of cytochrome P-450 2E1, which is called an alcohol-induced homogeneous compound that decomposes with ROS in the liver tissue of ASH patients and leads to 1-hydroxymethyl radicals from mitochondria, increases significantly [111,112]. The progression of fibrosis into cirrhosis can lead to an increased risk of developing HCC. However, despite the existence of cirrhosis, metabolic problems such as type 2 diabetes or insulin resistance increase the risk of hepatocellular carcinoma in individuals with nonalcoholic fatty liver disease (NAFLD).

## 8. Herbal Medicine Targeting Mitochondria-Mediated Oxidative Stress and Reactive Oxygen Species (ROS) in Liver Disease

In several experimental and clinical trials, herbal medications with anti-oxidative stress and lipid-balancing abilities have been used as pharmacological treatments for liver diseases. Growing evidence suggests that many natural medicines are involved in controlling lipid accumulation processes, including hepatic lipolytic and lipogenic pathways, such as mitochondrial and peroxisomal β-oxidation, the release of VLDL, the uptake of non-esterified fatty acids (NEFA), and some crucial hepatic lipogenic enzymes such as alanine amino transferase (ALT), and high density amino transferase (AST) [113,114,115,116]. The liver, mammary gland, and, to a lesser degree, adipose tissue produces them from two carbon units (acetyl-CoA). FFA (either saturated or unsaturated) are the form in which fat is transferred from adipose tissue to the sites of use. FFA circulate largely with albumin and serve a crucial role in delivering energy to the body, particularly during fasting. In people with central obesity, insulin resistance, and type 2 diabetes, FFA levels increase in the blood [72,117]. Notably, the degree of TG deposition predicts the severity of later stages of NAFLD, fibrosis, and cirrhosis. In order to avoid the advance of NAFLD and alleviate insulin resistance, inflammation, and oxidative stress, it has been demonstrated that enhancing hepatic lipid metabolism and reducing visceral fat have therapeutic potential [118]. 

*Alisma orientalis* induce fatty acid β-oxidation via activating lipid antioxidant enzymes such as carnitine palmitoyltransferase-1 (CPT-1) and lowing peroxidation. The mRNA and protein levels of fatty acid synthase (FASN) and acetyl-CoA carboxylase 1 (ACC1) were reduced following *Alisma orientalis* extract (AOE). The expression of the proteins Bcl-2-associated X protein (Bax), c-Jun N-terminal kinase (JNK), p-JNK (activated form of JNK), Bax, cleaved caspase-9, and caspase-3 were reduced. Following AOE therapy, the level of the anti-apoptotic B-cell lymphoma 2 (Bcl-2) protein was enhanced. In addition, AOE decreased inflammatory protein production, including p-p65, p65, cyclooxygenase-2 (COX-2), and inducible nitric oxide synthase (iNOS) [116,119]. 

The effects of the triterpenic acids-enriched fraction from *Cyclocarya paliurus* (CP) on NAFLD were examined. CP dramatically decreased malondialdehyde (MDA) and protein carbonyl (PCO) levels in Wister rats fed a high-fat diet. It also considerably boosted superoxide dismutase (SOD) activity and glutathione/oxidized glutathione (GSH/GSSG) ratio. Additionally, CPT increased nuclear factor erythroid 2-related factor 2 (Nrf2) and Nrf2-mediated antioxidant enzyme heme oxygenase1 (HO-1) production and repaired the malfunctioning of the mitochondrial membrane potential (MMP). In HepG2 cells exposed to free fatty acids, CPT markedly reduced ROS concentration while raising levels of the mitochondrial enzymes NADH dehydrogenase (Complex I) and cytochrome C oxidase (CCO). Additionally, CPT might boost the expression of HO-1, quinine oxidoreductase 1 (NQO1), and Nrf2 translocation from the cytoplasm to the nucleus. The findings showed that CPT might activate Nrf2 to protect mitochondrial function and enhance oxidative stress. As a result, it may be assumed that CPT might be a possible treatment for NAFLD [120].

Alcohol abstinence is crucial in NAFLD since even moderate alcohol use is connected with the advancement of liver fibrosis [121]. Through fibrosis, hepatic inflammation drives lipid buildup, redistribution, and liver damage from adipose to the liver, resulting in NAFLD [122]. Improvement of hepatic inflammation-mediated fibrosis is essential for the treatment of NAFLD, and the effect of herbal medicines on the inhibition of progression to fatty hepatitis has been confirmed. Furthermore, it has been shown to control dyslipidemia and improve liver function in NAFLD by inhibiting inflammatory signaling pathways. Many herbal medicines (including herbal milk powder, crude extracts and pure bioactive compounds from herbal medicine) such as Sinai san decoction, and *Hugan qingzhi* tablets have anti-inflammatory properties leading to improvement of NAFLD progression, including the reduction of liver inflammatory cytokines TNFα, interleukin-6 (IL-6), interleukin-1 beta (IL-1 β) [123,124,125,126,127,128]. 

*Lonicera caerulea* L. Polyphenol (LCP) reduces intestinal permeability glucagon-like peptide-2 content and occludin protein increase, whereas claudin-2 protein decreases), intestinal inflammation (levels of pro-inflammatory cytokines, such as TNF-α, IL-6, COX-2, and nuclear factor kappa B p65 (NF-κB p65) decrease, and intestinal ocular surface disease (*OSD*). In addition, LCP reduces LPS-induced liver damage by inhibiting nuclear translocation of NF-κB p65 and activation of the mitogen-activated protein kinase (MAPK) signaling pathway [129]. 

*Polygonum multiflorum* has two medicinal forms, *Polygoni multiflori radix* and *Polygoni multiflori radix prapaerata*. Notably, there is an increasing interest in whether *Polygonum multiflorum* has a hepatotoxic impact or not. Both forms have the same therapeutic efficacy against NAFLD, fibrosis, and cirrhosis when the daily consumption is less than 6g per individual [130,131,132]. The important mechanism of hepatotoxicity for both forms may include cell cycle arrest and enhance the activities of alanine aminotransferase (ALT), aspartate aminotransferase (AST), alkaline phosphatase (ALP), creatinine, total bilirubin (TBil), direct bilirubin (DBil), and indirect bilirubin (IBil), as well as the leakage of LDH, whereas cytochrome P3A4 (CYP3A4) and cytochrome P2C19 (CYP2C19) drug metabolic enzymes do not [130,133,134,135].

Gypenosides were considerably elevated mRNA and protein levels of sterol regulatory element-binding protein (SREBP)-1c and carbohydrate responsive element binding protein (ChREBP) in the liver tissue homogenates of high-fat diet-induced rat NASH models. Stearoayl desaturase (SCD-1), lipogenic enzymes, and prolonged activation of SREBP-1c contribute to the development of fatty liver disease and dyslipidemia [136,137]. 

Adipocyte histopathology, hepatocyte hypertrophy, hepatic enzyme activity, lipid metabolism, and associated gene expression, including ACC1, AMPK 1 and AMPK 2 in hepatic tissue, and leptin, UCP2, adiponectin, C/EBP, C/EBP, and SREBP-1c in adipose tissue, were all enhanced by Korean blue honeysuckle (BH). BH extract consistently reduced the risk factors for NAFLD and obesity through AMPK upregulation-mediated hepatic glucose enzyme activity, lipid metabolism-related gene expression, and activation of the antioxidant defense system [138].

The expression of SREBP-1c and its target genes is markedly elevated in the livers of NAFLD patients. ChREBP is a transcriptional activator of lipogenic and glycolytic genes and a major regulator of hepatic de novo fatty acid production under healthy settings and in NAFLD [139,140]. It was confirmed that *Nuclear factor*-κB (NF-κB) signals and *Lycium barbarum* polysaccharides identified in monocytic chemotactic protein-1 (MCP-1) inhibition, macrophage inflow, and decreased hepatocellular apoptosis, and moreover, NF-κB signals were suppressed and decomposed caspaces-3 [113] 

Total alkaloids in *Rubus aleaefolius* Poir (TARAP) is a traditional Chinese medicine that has long been used to treat NAFLD abroad. In NAFLD rats, it was discovered that TARAP could lower blood levels of TG, total cholesterol (TC), and low-density lipoprotein (LDL-C) and raise serum levels of HDL-C. Additionally, TARAP therapy elevated the expression of carnitine palmitoyl transferase and downregulated the expression of fatty acid synthetase (FAS) and acetyl-CoA carboxylase (ACC) (CPT) [141]. One of the oldest and most popular botanicals in traditional eastern medicine is Korean red ginseng (*Panax ginseng* Meyer). For its capacity to lengthen life and boost vitality and longevity, Korean red ginseng extract (RGE) is advised. Korean red ginseng is *P. ginseng* that has undergone a heat-processing procedure to increase its pharmacological and biological effects [142]. In particular, ginsenosides Rb1, 25-OCH3-PPD, and Rg1 from *P. notoginseng* have been shown to suppress hepatic stellate cells (HSC) activation and promote their apoptosis [143,144,145]. RGE treatment significantly reduced TGF-β1, PAI-1, and immunohistochemistry of alpha-smooth muscle actin (α-SMA), one of the characteristic HSC transactivation indicators [145].

**Figure 5:**
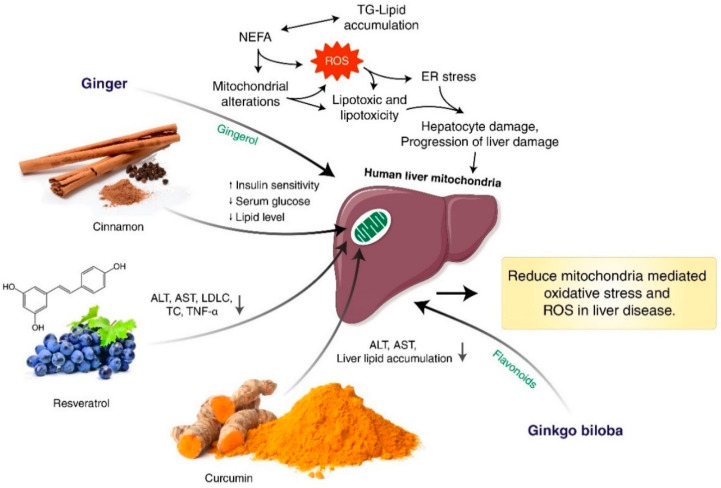
Effects of herbal compounds to reduce mitochondria-mediated oxidative stress and ROS in liver disease. Alanine amino transferase (ALT), and high density amino transferase (AST), non-esterified fatty acids (NEFA), low-density lipoprotein cholesterol (LDLC), total cholesterol (TC), tumor necrosis factor alpha (TNF-α). The accumulation of TG and NEFA induces mitochondrial deformation and ROS, resulting in liver and hepatocyte damage caused to lipotoxicity and ER stress. Cinnamon improves insulin sensitivity, decreasing lipid and blood glucose. Low-density lipoprotein cholesterol (LDLC), total cholesterol (TC), and TNF-α decreased by resveratrol. Curcumin reduced AST and ALT levels and liver lipid storage. ↑, up-regulation; ↓, down-regulation.

Sophocarpine (derived from foxtail-like sophora herb and seed) lowered serum aminotransferase and total bilirubin levels in rats subjected to continuous stress. Furthermore, sophocarpine inhibited extracellular matrix deposition and reduced the development of hepatic fibrosis. In addition, sophocarpine suppressed the expression of α-SMA, interleukin (IL)-6, transforming growth factor-1 (TGF-β1), and toll-like receptor 4 (TLR4) [146]. Sophocarpin is known to contribute to anti-NASH effects via AMPK, a major regulator of cellular energy balance as a master switch of glucose and lipid metabolism in a variety of organs, including skeletal muscle and the liver [147]. ER stress plays a role in the progression of NAFLD and pathogenesis of NASH, and activation of farnesoid X receptor (FXR) by betulinic acid-alleviated liver stress-mediated HS. Betulinic acid acts as an FXR that attenuates the formation of HFD and MCD-induced NAFLD, and it has been confirmed that *Allisma orientalis* stimulates FXR activation, especially allisol A24B-acet action, thereby restoring hepatocellular ER homeostasis [148]. Naringgenin, ginsenoside Rb1, and *Leonurus japonicus* Houtt extract, which recruit insulin receptor substrate-1 (IRS-1), activate PI3K/Akt to induce protein kinase A (PKA) and serum and glucocorticoid kinase *3* (SGK-3β), ultimately promote glycogen and lipolysis synthesis, and inhibit hyperinsulinemia and NAFLD [149]. *Citrus* polymethoxylated flavones (PMF) also reduced TG contents in the liver and heart and were able to regulate adipocytokines by significantly suppressing TNF-α, TNF-γ, IL-1β and IL-6 expression and increasing adiponectin in IR. The mechanism of PMF on PPAR activation was also investigated, and PPAR and PPAR protein expression were shown to be dramatically elevated in the liver [150]. Yin-Chen-Hao decoction (YCHD), for example, has the active component scoparone, which has been used clinically in traditional Chinese medicine formulations for over a thousand years to treat hepatic dysfunction, cholestasis, and jaundice [151]. YCHD demonstrates protective effects against an experimental model of liver fibrosis by inhibiting the activation of HSCs [152]. Other fibrosis-related metabolites such as unsaturated fatty acids and lysophosphatidylcholines (Lyso-PCs) were among the seven found to have significantly changed. Because YCHD inhibits oxidative stress and the lipid peroxidation it induces, both of which are linked to hepatic fibrogenesis, it may be the reason why it possesses anti-fibrotic characteristics [153]. Nobiletin (NOB) is a polymethoxylated flavone found in citrus fruits as *Citrus depressa*, *C. sinensis* (oranges), and Limon. NOB, also known as 5,6,7,8,3,4-hexamethoxyflavone, is a flavonoid [154,155,156]. Numerous biological effects of NOB, including antioxidant, free radical scavenger, anti-inflammatory, anti-tumor, lipid-lowering, and insulin-sensitizing capabilities, have been demonstrated [154,155,157,158]. NOB reduced NASH progression and fibrosis via regulating hepatic oxidative stress and reducing mitochondrial dysfunction [156]. Ursolic acid (UA) is a naturally occurring ingredient that has been demonstrated to have antifibrotic properties and is present in a range of plants. By reducing the activity and expression of NOX/ pyrin-domain-containing 3 (NLRP3) inflammasome signaling, UA suppresses HSC activation and reverses liver fibrosis [159]. UA were found to have the effect of improving insulin resistance and amplifying glucose absorption through IRS-1/AKT stimulation in NAFLD treatment. *Shenling baizhu* powder was found to relieve hepatic steatosis and protect colon mucosa due to decreased expression of endotoxin and inflammatory media (TNF-α, IL-1β) through the TLR4 pathway, and diamond glycyrrhizic acid was proven to reduce intestinal inflammation and restore barriers [128,160,161,162]. Few NAFLD treatments target distinct pathways and may be effective against dysfunctional mitochondria. Antioxidants that target mitochondria are one potential method for addressing NAFLD-associated liver diseases. Our review indicates that herbal medicine inhibited NAFLD progression and fibrosis through regulating hepatic oxidative stress and reducing mitochondrial dysfunction. Herbal medicine may therefore be an unique and promising therapy for NAFLD and liver fibrosis. The effects of herbal substances on reducing oxidative stress and reactive oxygen species in liver disease caused by mitochondria presented in Figure 5. As a result of its ability to block growth factors including TGF and vascular endothelial growth factor (VEGF), induce apoptosis, and regulate MAPK pathways, naringenin offers protection against the development of HCC [163,164]. It has been demonstrated beyond a reasonable doubt that silymarin is effective in inhibiting OS; hence, its utilization is advised for the treatment of ALD and NAFLD [165]. Through modulation of the TNF-alpha/NF-kappaB signaling pathway, L-theanine protects C57BL/6J mice from developing acute alcoholic liver damage [166]. Hesperidin and myricetin are flavonoids with anti-inflammatory and anti-oxidant properties, and both of these flavonoids have been shown to be helpful in the treatment of fatty liver disease (FLD) [167]. In human fetal immortalized hepatocytes, caffeine causes disruption in gene-related pathways that are associated with ataxia telangiectasia and exacerbates the toxic effects of acetaminophen [168]. Quercetin suppressed liver inflammation through NF-B/TLR/NLRP3, reduced PI3K/Nrf2-mediated oxidative stress, activated mTOR in autophagy, and inhibited apoptotic markers associated with liver disease [169,170].

## 9. The Antioxidant Effect of Herbal Medicines via Suppression of Lipid Peroxidation to Thiolation Migration in Oxidative Damage

Herbal medicines feature anti-inflammatory, antioxidant, liver-protective, and anti-cancer properties, and they can prevent liver damage caused by a variety of conditions [171]. Flavonoids, which are abundant in herbal medicines, are distinguished by their antioxidant properties. Flavonoids’ structural properties and antiradical activities are inextricably linked [172]. Flavonols and flavones belong to a wide category of polyphenolic flavonoids renowned for their antioxidative properties [173]. The 5-OH group is among the most widespread hydroxyl groups in flavonoids and may be present in several flavonoids including chrysin galangin, apigenin, luteolin and morin. Intramolecular hydrogen-bond (IHB) is well regarded between 5-OH and the C4=O keto group, the antiradical capability of 5-OH as hydrogen atom extraction from 5-OH requires additionally breaking the H5⋯O=C4 IHB [174]. Recently, employing density functional theory (DFT) based on radical scavenging processes including hydrogen atom transfer (HAT), single electron transfer-proton transfer (SET-PT), and sequential proton-loss electron-transfer (SPLET), it has been identified that the effect of the H5⋯O=C4 intramolecular hydrogen-bond (IHB) on the antiradical activity of flavonoid was disclosed. The thermodynamic parameters of these processes were determined, including bond dissociation enthalpy (BDE), ionization potential (IP), proton dissociation enthalpy (PDE), proton affinity (PA), and electron transfer enthalpy (ETE). It indicated that the H5⋯O=C4 IHB has the critical role on the 5-OH group, and its antiradical potential is decreased. Notably, it was determined that the H5⋯O=C4 IHB has the greatest effect on the 5-OH group, consequently diminishing its antiradical capability. H5⋯O=C4 IHB would weaken flavonoid antiradical action by raising the bond dissociation enthalpy [172]. In addition, highly active flavonoids often have a catechol moiety, the activity of which was recently established for additional families of polyphenolic compounds [175,176,177]. The C2–C3 double bond extends π-conjugation onto the carbonyl group in the C-ring; hence, the radical scavenging capacity of unsaturated flavonoids is larger than that of saturated structures, such as flavanones [178]. This study underscores the importance of catechol moiety, and several studies indicate that it can play a vital role in reducing its possible side effects [179,180,181,182]. Antioxidant medicine can be utilized to alleviate diseases spurred on by oxidative stress. The catechol moiety found in several antioxidants, including catecholamines and numerous flavonoids, is a crucial antioxidant pharmacophore [183]. A monoamine neurotransmitter called a catecholamine is an aldehyde or a ketone having a catechol (benzene with pair hydroxyl side groups) and a side-chain amine [184]. They can eliminate highly reactive species, such as peroxynitrite and the hydroxyl radical [179,185]. During this reaction, the antioxidant is transformed into semiquinone radicals and quinones, which are oxidized products. These components may also be hazardous [186,187,188]. Recent studies have shown the effect catechol-containing antioxidants have on free group damage. To investigate the effects of catechol-containing antioxidants, 4-methyl-orto-benzoquinone, a stable oxidation product, was adopted [189,190]. The capability of 4-methylcatechol to reduce microsomal lipid peroxidation demonstrates that the catechol moiety is a powerful antioxidant pharmacophore [183,191]. This finding implies that the oxidation products of catechol-containing antioxidants transfer the oxidative stress-induced damage from lipid peroxidation to sulfhydryl arylation. Deactivating the endogenous defenses against lipid peroxidation, i.e., the GSH-dependent free radical reductase, is one of the potential side effects of this sulfhydryl arylation. This indicates that despite the direct protection provided by catechol-containing antioxidants, lipid peroxidation is indirectly increased by the reaction products of these antioxidants generated during this protection. One of the principal harmful consequences of lipid peroxidation is calcium ATPase inhibition. Antioxidants including catechol reduce lipid peroxidation, however the reactive chemicals generated during this protection impede calcium ATPase as well. So, despite the apparent protection against lipid peroxidation provided by catechol-containing antioxidants, the harmful impact on a final target, calcium ATPase, is the same [192]. Their antioxidative ability was found to be highly dependent on their molecular structure and substitution pattern: the availability of hydroxyl groups. As previously stated, their antioxidant behavior cannot be fully explained until interactions with the surrounding media are considered. This is especially true in complex biological contexts, where, in addition to water, a diversity of H-bonding ligands might be employed to control antioxidant reactivity. As a result, it is critical that they keep their prescribed integrity [175,179,193,194,195,196,197].

## 10. Drug Target and Clinical Use of Herbal Medicine to Reduce Mitochondria-Mediated Oxidative Stress

The usefulness of antioxidant potential for the treatment of liver diseases is due to a molecular imbalance between ROS and antioxidants. It has been noted that the balance between GSH/GSSG and cysteine/cystine oxidation reaction and antioxidant defense has a cysteine concentration relationship, but is not related to cystine of GSSG [198]. The effect of GSH affects both NAFLD and ALD, suggesting that increased production of ROS and prooxidants is directly related to disease progression and acts to inhibit mitochondrial antioxidant defense [199,200]. Although administration of antioxidant cocktails of vitamin E and NAC did not improve the survival rate of the AH patient cohort, interestingly, GSH levels are supplemented by NAC or S-adenosylmethionine, showing increased efficacy of prednisolone in ALD patients, as the ‘S’-adenosylmethionine donor act as GSH precursor and targets multiple hepatocyes [201,202,203].

A chemical SOD mimetics method of natural SOD enzymes has been developed to overcome the intracellular immune reaction side effects of natural SOD enzymes [204,205]. Accordingly, it was observed that manganese (III) mesotetrakis (N-ethylpyridinium-2-yl) porphyrin MnP is effective in liver steatosis and HFD-induced obesity [206]. MnP is known as first redox enzyme, which has anti-inflammatory properties by superoxide scavenging and targeting the nuclear factor kappa B [95]. MnTBAP was found to prevent liver lipid accumulation and prolong lifespan due to the substitution of SOD2 deficiency in Sod2^tm1Cje^ null mice. These NAFLD models induced mGSH depletion, leading to increased mGSH levels with GSH ethyl ester (GSHEE) by MnTBAP, resulting in the production of GSH of MnTBAP effects [6,95]. Therefore, these results show that it is necessary to maintain mGSH in antioxidant balance against antioxidant stress due to SOD2 and NAFLD progression. The role of SOD mimetics in ALD may vary with mGSH, which demonstrated exacerbation of mtDNA depletion in SOD2-deficient mice [207,208]. 

**Figure 6:**
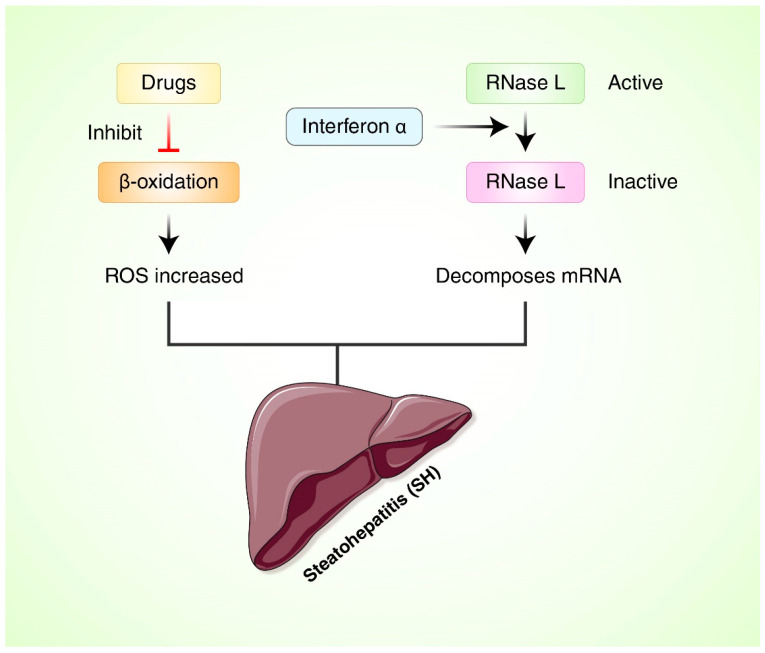
Drug target to reduce oxidative stress in liver damage. *Ribonuclease L* (RNase L), interferin.

Some synthetic drugs have targeted mitochondrial-damaged cause steatohepatitis, either inhibiting β-oxidation or depleting their cofactors, or directly inhibiting replication and transcription of MRC complexes and mtDNA, and others induce mtDNA damage due to increased ROS [30]. Diethylaminoethoxyhexestrol, perhexiline, amiodarone, and tamoxifen are examples of drugs that inhibit β-oxidation [31,209,210]. Several mitochondrial hepatotoxic drugs include inhibiting mitochondrial β-oxidizing such as tetracycline, 2-arylpropion, aminectine, perhexylin, and tamoxipene, and can also inhibit electron transfer in MRC [30,31] (Figure 6). Interferon alpha, a treatment for patients with chronic HBV infection, changes translation with mitochondrial transcription to activate *Ribonuclease L* (RNase L), which decomposes TFAM messenger RNA and mtDNA encoded mRNA [30,211]. Diabetes can increase the risk of liver failure due to acute drugs and obese women can increase fatty hepatitis caused by tamoxifen. Obese patients with rheumatoid arthritis can cause liver damage when methotrexate is administered [212,213,214]. In a randomized clinical trial of NAFLD, herbal medicine was found to be effective as a way of normalizing AST and causing the disappearance of radiological steatosis in patients [215]. Consumption of resveratrol for 12 weeks showed significant effectiveness, and decreased insulin-resistant ALT, AST, low-density lipoprotein cholesterol (LDLC), TC, and TNF-α were found in a NAFLD patient, but further confirmation and investigation of adverse effects, further efficacy and safety demonstration were required [216]. It has been demonstrated that a randomized placebo-controlled curcumin trial showed decreased liver lipid accumulation and AST and ALT levels in NAFLD patients without resistance [217]. It was also investigated whether cinnamon acts as insulin sensitization through improved serum glucose and lipid levels in people with non-insulin-dependent type 2 diabetes and NAFLD patient studies [218]. 

## 11. Conclusions and Perspectives

Recent studies have shown that oxidative stress is always a contributing factor in progressive liver disease. This type of oxidative stress is especially activated in hepatocytes and specific pro-oxidant herbal medicine, regulating the introduction of potentially hazardous stress in order to successfully trigger oxidative hepatotoxicity [219]. This is the case despite the fact that the liver is equipped with a well-established defensive system to protect hepatocytes from oxidative damage. NAFLD and ALD are the main keys of molecular mechanisms and mitochondrial-mediated oxidative stress process in liver. However, due to the complex task, diverse metabolic reactivity takes place only in complex steps, that depend on DNA, protein, and lipids. It is advantageous to inhibit the production of free radicals with antioxidants, but their association with human diseases has not yet been identified [7]. Therefore, imbalance of the dual function of ROS/oxidative stress contribute to mitochondrial dysfunction, causing disease progression [220]. Unfortunately, ROS are not yet considered important for cell pathophysiology, which may play a role in regulation acting in association with disease and aging by upregulation of the antioxidant mechanism. In both NAFLD and ALD, SOD mimetics in an experimental model produce more harmful ROS, such as hydroxyl radicals, as powerful oxidants, in mGSH and mitochondrial antioxidant defense, which failed despite the decrease in superoxide anion [7,221]. Numerous herbal medicines have significant bioactivity with less cytotoxicity and adverse effects than synthesized medications, owing to their vast structural and chemical diversity. New therapeutic agents generated from natural products are required to treat liver diseases and their consequences with fewer adverse effects than those induced by present drugs. Additionally, it is possible that hepatic metabolic dysregulation is the primary pathogenic mechanism implicated in herbal medicine-induced hepatotoxic impairment. Thus, practitioners should be aware of hepatotoxic dangers before utilizing herbal medicine. The restricted findings in this research for many disorders without hepatotoxicity should also be researched in further studies.
